# Optimal alpha-1 antitrypsin level cutoffs for genotype identification in patients with chronic liver disease

**DOI:** 10.1097/HC9.0000000000000023

**Published:** 2023-01-20

**Authors:** Sameer Prakash, Arvind R. Murali

**Affiliations:** 1 University of Iowa Hospitals & Clinics, Iowa City, Iowa, USA; 2 Memorial Hermann Health System, Houston, Texas, USA; 3 Orlando Health, Orlando, Florida, USA

## Abstract

Controversy exists whether alpha-1 antitrypsin (A1AT) genotype testing should be performed as a first-line screening for A1AT heterozygous variants.

**Methods:**

We calculated the median and interquartile range of A1AT level for each genotype in 4378 patients with chronic liver disease and “miss rate” of MZ genotype identification at various cutoff levels.

**Findings:**

Significant overlap in A1AT level noted with Pi*MM, MZ, and MS variants. Miss rate of Pi*MZ at a cutoff level <100 was 29%, <110 was 18%, <120 was 8%, and <130 was 4%. We suggest simultaneous measurement of A1AT level and genotype in patients with chronic liver disease.

## Graphical abstract

**Figure:**
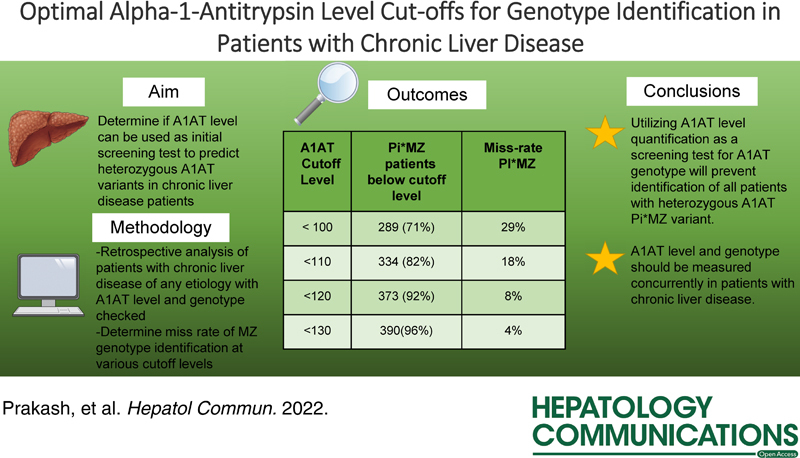

## INTRODUCTION

Alpha-1 antitrypsin (A1AT) deficiency is a common but an underdiagnosed genetic disorder. The homozygous A1AT Pi*ZZ phenotype is associated with chronic liver disease and cirrhosis. More recently the heterozygous A1AT Pi*MZ phenotype has been recognized as an important disease modifier in patients with metabolic syndrome–associated fatty liver disease, and alcohol-associated fatty liver disease.^1^ Studies have shown that heterozygous A1AT Pi*MZ phenotype increases the risk of developing cirrhosis and hepatic decompensation in patients with metabolic syndrome–associated fatty liver disease,^1^–^3^ It is thus essential to determine if a patient with chronic liver disease carries the Pi*MZ phenotype. Serum A1AT level is commonly ordered as part of the initial evaluation for chronic liver disease and is often used as a surrogate for A1AT genotype.^4^,^5^ However, A1AT is an acute phase reactant, and its level can vary with hepatic and systemic inflammation. In addition, there is a significant overlap in the A1AT level among the various genotypes. We thus aimed to determine if the A1AT level can be used as an initial screening test to predict heterozygous A1AT variants in patients with chronic liver disease.

## METHODS

We retrospectively reviewed the records of patients with chronic liver disease of any etiology who had both A1AT level and genotype checked at a single large tertiary academic center, the University of Iowa Hospitals & Clinics from 2005 to 2020. A database of patients with chronic liver disease who also had A1AT genotyping done was identified using the *International Classification of Diseases, 10th Revision* (ICD-10) codes for various liver diseases including alcohol-associated liver disease, NAFLD, NASH, autoimmune liver diseases, chronic hepatitis B, chronic hepatitis C infection, abnormal liver enzymes, and any cirrhosis. Patients without A1AT genotyping were excluded. The method used to measure A1AT level was immunoturbidimetric assay. We calculated the median, interquartile range (IQR), and range of A1AT level for—Pi*MM, MZ, MS, SS, SZ, SS, and ZZ genotypes. We then calculated the “miss rate” of MZ genotype identification at various cutoff’s of A1AT level, if the A1AT level was used as an initial screening test for A1AT genotype determination. Miss rate was defined as the number of patients with MZ phenotype who would not have been identified when a particular A1AT cutoff was used for additional genotype testing. All research was conducted in accordance with both the Declarations of Helsinki and Istanbul and approved by the University of Iowa Institutional Review Board. Our research received a waiver of informed consent.

## RESULTS

We included 4378 patients in this retrospective cohort study (Suppl Table 1, http://links.lww.com/HC9/A79). Pi*MM was seen in 3538 patients (80.8%), 341 had Pi*MS (7.8%), 407 Pi*MZ (9.3%), 8 Pi*SS (0.002%), 35 Pi*SZ (0.8%), and 49 Pi*ZZ (1.1%). The median (IQR) A1AT level for each genotype were Pi*MM 150 (132–174), Pi*MS 127 (112–147), Pi*MZ 91 (80–103), Pi*SS 102 (93–112), Pi*SZ 59 (49–76), and Pi*ZZ 29 (<20–34). There was a significant overlap in the range of A1AT level among the A1AT genotypes (Suppl Table 2, http://links.lww.com/HC9/A80). The miss rate of MZ phenotype at a cutoff A1AT level of <100 was 29%, at <110 was 18%, at <120 was 8%, and for <130 was 4% (Table 1). Conversely, at an A1AT level cutoff of <130, 23% had Pi*MM genotype (Table 1).

**TABLE 1:** Miss rate of Pi*MZ and Pi*MM genotypes at various A1AT cutoff levels

A1AT cutoff level	No, patients with MZ below the cutoff (total N=407) [n (%)]	Miss rate of Pi*MZ (%)	No. PI*MM patients below cutoff (total N=3538) [n (%)]
<100	289 (71)	29	22 (0.6)
<110	334 (82)	18	148 (4)
<120	373 (92)	8	404 (11)
<130	390 (96)	4	820 (23)

Abbreviation: A1AT, alpha-1 antitrypsin.

There was no significant difference in the Pi*MZ A1AT level among the various etiologies of liver disease. The median (IQR) of A1AT level in MZ patients with alcohol-associated liver disease, NAFLD, chronic viral hepatitis, and other etiologies was 99 (33.5), 93 (23.3), 94 (24.0), and 87 (22.0) respectively. Similarly, the mean (SD) A1AT level in MZ patients with alcohol-associated liver disease, NAFLD, chronic viral hepatitis, and other etiologies was 103 (28.8), 95 (21.8), 96 (19.8), and 95 (32.4) respectively.

We calculated the median (IQR) A1AT level for MM and MZ patients stratified by FIB-4 index (for FIB-4 <1.45 and FIB-4 >3.25). For Pi*MM patients, the median (IQR) A1AT level for was 144 (128–167) for FIB-4 <1.45 and 163 (143–185) for FIB-4 >3.25. For Pi*MZ patients, the median (IQR) A1AT level was 92 (81–104) for FIB-4 <1.45 and 97 (81–108) for FIB-4 >3.25. Overall, the A1AT level in A1AT MZ patients was similar stratified by the FIB-4 index.

## DISCUSSION

The cutoff level to identify heterozygous A1AT variants remains controversial. Simsek et al.^6^ determined that an A1AT cutoff value of 100.5 mg/dL had a negative predictive value of 99.9% to detect Pi*MZ in a healthy Turkish population but a positive predictive value of just 5%. Kok and colleagues showed that the A1AT level was above 100 mg/dL in a significant number of Pi*MZ patients with chronic liver disease of varying etiologies. In addition, they showed that the A1AT level was >100 mg/dL in 83% of Pi*MZ patients with A1AT deficiency-associated cirrhosis, thus suggesting that underlying liver inflammation can falsely elevate the A1AT level as part of the acute phase response.^7^. In a study by Bornhorst et al.^8^, the percentage of Pi*MZ samples at a cutoff level of 100, 110, 120, and 130 were 69.4%, 82.4%, 89.5%, and 94.1%, respectively. In a large population study of patients enrolled in the Swiss Cohort Study on Air Pollution and Lung Diseases in Adults (SAPALDIA), Ferrarotti et al.^9^ found narrower serum AAT ranges compared with other studies and described a cutoff level at 110 mg/dL to distinguish homozygous Pi*MM from heterozygous Pi*MZ and Pi*SZ alleles. In our study, we have shown that in patients with chronic liver disease of varying etiologies, using A1AT level as a surrogate for A1AT genotype would risk missing identification of Pi*MZ phenotype in a significant number of patients. Using an A1AT level cutoff of 130 or higher would decrease the “miss rate” but would substantially increase the number of patients (∼25% at a cutoff of 130) subjected to repeat blood draw for genotype analysis. Snyder et al.^10^ have previously proposed simultaneous testing of A1AT level and genotype assay for patients with suspected A1AT deficiency-associated liver disease in their diagnostic algorithm. However, this has not yet been adopted in most clinical practices. An important limitation of our study is that our study cohort is from a single large academic institution with a predominantly Caucasian population (86.4%) and thus these results may not be applicable to all populations.

In conclusion, our study shows that utilizing A1AT level quantification as a screening test for A1AT genotype will prevent identification of all patients with heterozygous A1AT Pi*MZ variant. Given the importance of A1AT Pi*MZ phenotype in the prognostication of patients with chronic liver disease, and the variability of the A1AT level in the setting of hepatic and systemic inflammation, we suggest simultaneous measurement of the A1AT level and genotype in patients with chronic liver disease.

## Supplementary Material
