# Supplementary material for: Optimal alpha-1 antitrypsin level cutoffs for genotype identification in patients with chronic liver disease
Source: Hepatol Commun. 2023 Jan 20;7(2):e0023. doi: 10.1097/HC9.0000000000000023 (PMC10019232; doi:10.1097/HC9.0000000000000023)
Supplement: Supplementary file 1 [file hc9-7-e0023-s001.doc]

| Etiology | Number of patients, n |
| --- | --- |
| Alcohol associated liver disease | 914 |
| Metabolic syndrome associated fatty liver disease | 2520 |
| Viral hepatitis | 450 |
| Others | 1677 |

*Others include autoimmune hepatitis, PBC, PSC; vascular disorders such as Budd Chiari syndrome; hemochromatosis; and unknown etiology.

Suppl Table 1: Etiologies of chronic liver disease among patients included in the study
