# Supplementary material for: Optimal alpha-1 antitrypsin level cutoffs for genotype identification in patients with chronic liver disease
Source: Hepatol Commun. 2023 Jan 20;7(2):e0023. doi: 10.1097/HC9.0000000000000023 (PMC10019232; doi:10.1097/HC9.0000000000000023)
Supplement: Supplementary file 2 [file hc9-7-e0023-s002.docx]

| Genotype | Number of Patients (n = 4378) | Median A1AT Total Level (IQR) | A1AT Level Range |
| --- | --- | --- | --- |
| MM | 3538 | 150 (132-174) | 83-463 |
| MS | 341 | 127 (112-147) | 66-295 |
| MZ | 407 | 91 (80-103) | 20-218 |
| SS | 8 | 101.5 (92.5-111.5) | 85-131 |
| SZ | 35 | 58.5 (49-75.75) | 30-140 |
| ZZ | 49 | 29 (25-33.5) | 21-83 |

Suppl Table 2: Median A1AT Level, IQR, and range of A1AT level among genotypes
